# Bisphosphonate-Related Osteonecrosis of the Jaw: Historical, Ethical, and Legal Issues Associated With Prescribing

**Published:** 2013-01-01

**Authors:** Beth Faiman, Aiswarya Lekshmi Pillai Chandran Pillai, Ana Gabriela Benghiac

**Affiliations:** From Cleveland Clinic Foundation, Cleveland, Ohio; Case Western Reserve University, Cleveland, Ohio; and St. Spiridon Hospital, Iasi, Romania

## Abstract

The long-term effects of many drugs are unknown. Established risks are communicated to patients who participate in clinical trials during the informed consent process. However, unknown and unanticipated side effects of medications may occur years after treatment. Patients with metastatic bone cancer experience an imbalance between tumor cells and the bone marrow microenvironment. Increased cytokine release, osteoclastic activity, and uncoupled osteoblastic activity lead to weakened bone structure and osteolytic lesions. The bisphosphonates are a class of drugs available in IV and oral formulations to treat and prevent bone loss and decrease the risk of skeletal-related events. Intravenous bisphosphonates such as zoledronic acid and pamidronate disodium are approved by the US Food and Drug Administration for the treatment of bone pain and hypercalcemia of malignancy and the prevention of painful bone fractures in patients with metastatic bone cancer. Oral bisphosphonates such as alendronate, risedronate, and etidronate are used to reduce the risk of skeletal fractures in patients with osteoporosis and in breast cancer. Bisphosphonate-related osteonecrosis of the jaw (BRONJ) is a rare but painful complication of treatment characterized by infection, exposed bone, and poor wound healing. In this article, we discuss BRONJ and identify past, present, and future ethical and legal issues surrounding bisphosphonate administration.

There are several synonyms for bisphosphonate-related osteonecrosis of the jaw (BRONJ). Some authors have described the phenomenon as bisphosphonate-associated osteonecrosis of the jaw, bisphosphonate-associated osteonecrosis, osteochemonecrosis of the jaws, and "bis-phossy jaw." The term bis-phossy jaw has historical implications, as it is correlated with the early nineteenth century occupational hazard that was experienced by matchstick makers exposed to white phosphorus, referred to as "phossy jaw" (Marx, 2008).

Bisphosphonates, which were previously known as diphosphonates, are a class of synthetic compounds with a chemical structure similar to pyrophosphate (a regulator of bone mineralization) but more resistant to hydrolysis in acid conditions (Rogers et al., 2000; Sparidans, Twiss, & Talbot, 1998). The main role of bisphosphonates is the prevention of bone loss through the precipitation of calcium and phosphorus from the bone (Russell, Croucher, & Rogers, 1999). Although the mechanism of action is complex, these potent inhibitors of bone resorption effectively decrease serum calcium levels in patients with hypercalcemia of malignancy and prevent bone fractures in patients with metastatic bone cancer and osteoporosis (Licata, 2005). Bisphosphonates are also used in the management of metabolic bone diseases such as osteoporosis, Paget’s disease, fibrous dysplasia, metastatic cancers (prostate, breast, kidney, lung, liver, and breast), multiple myeloma, osteogenesis imperfecta, reflex sympathetic dystrophy, heterotopic ossification, and other bone conditions.

A primary role of these drugs is to reduce bone loss and inhibit bone turnover by suppressing the osteoclast activity (Raje & Roodman, 2011). Osteoclasts are responsible for bone breakdown and turnover, a process that is accelerated in patients with cancer. Bisphosphonates are prescribed widely in different doses and have a short half-life in circulation but can last in bone up to 10 years, depending on the bone turnover time.

Insight into the mechanics of bone turnover has led to the development of new therapies to prevent and treat cancer-related bone disease, including denosumab (Prolia, Xgeva). Denosumab is a receptor activator of nuclear factor kappa B ligand (RANKL) inhibitor and blocks the formation and activation of osteoclasts. When given as Prolia, denosumab is administered at a dose of 60 mg every 6 months. Prolia is approved by the US Food and Drug Administration (FDA) for the following indications: (1) the treatment of postmenopausal women with osteoporosis at high risk for fracture, (2) to increase bone mass in men at high risk for fracture (those with osteoporosis and those receiving androgen deprivation therapy for nonmetastatic prostate cancer), and (3) to increase bone mass in women at high risk for fracture receiving adjuvant aromatase inhibitor therapy for breast cancer (Amgen, 2010a).

Denosumab (Xgeva) is approved by the FDA for the prevention of skeletal-related events (SREs) in patients with bone metastases from solid tumors not related to multiple myeloma (Amgen, 2010b). When given as Xgeva, denosumab is administered at a dose of 120 mg every 4 weeks as a subcutaneous injection. Although generally well tolerated, denosumab has side effects similar to those of the bisphosphonates, such as acute phase reactions and renal insufficiency. Denosumab use can lead to osteonecrosis of the jaw. However, as denosumab does not belong to the bisphosphonate class of drugs, we will not review further ethical issues relating to denosumab in this article.

## Bisphosphonates: Historical Perspective

Bisphosphonates were first used in the chemical industry in the early 1900s for their antiscaling and anticorrosive properties (Ruggiero, 2009). Bisphosphonate trials in humans first took place in the 1960s (Chesnut, 1996). Bassett and colleagues (1969) reported the effect of bisphosphonates in three cases of patients with myositis ossificans, a rare, incurable, and potentially fatal childhood disease. One child (aged 16 months) was given oral disodiumethane-l-hydroxy-1,1-diphosphate, a form of bisphosphonate. As a result, she had improvement in respiratory function and lived into adulthood. Based on Bassett’s findings, bisphosphonate experimentation in human subjects became widespread. Little is known regarding the ethics and methodology of early studies, but bisphosphonates were first given to patients to treat Paget’s disease of the bone and osteogenesis imperfecta when no other treatment existed for these conditions (Licata, 2005).

Etidronate was the first oral bisphosphonate studied within the context of clinical trials (Watts et al., 1990). In a double-blind, placebo-controlled trial, 429 women with postmenopausal osteoporosis and at least 1 vertebral compression fracture as a result of osteoporosis received intermittent etidronate for 2 years. In this trial, oral etidronate was found to significantly increase spinal bone mass and reduce new vertebral fractures in women with postmenopausal osteoporosis (Watts et al., 1990).

Alendronate, risedronate, and ibandronate are oral bisphosphonates that have been shown to reduce the risk of fracture in postmenopausal women with osteoporosis. These agents were closely behind etidronate in clinical trials. More potent IV bisphosphonates such as pamidronate disodium and zoledronic acid (Zometa) were subsequently studied in patients with metastatic cancer and multiple myeloma to decrease the risk of SREs (Berenson et al., 1996; Body, 2003; Major & Coleman, 2001; Major et al., 2001). Since these earlier trials, thousands of individuals have participated in studies to determine the safety and efficacy of these compounds in humans.

## Benefits of Bisphosphonate Use

Short- and long-term benefits of bisphosphonate use have been described. Short-term benefits primarily exist in patients with hypercalcemia of malignancy. It is for this indication that bisphosphonates rapidly decrease life-threatening blood calcium levels and improve bone pain as a result of osteolysis, or bone damage. The primary long-term benefit for patients who take bisphosphonates is a reduction in one’s risk of developing SREs or bone fractures. The spine and vertebral column are particularly vulnerable to fracture. Weakened bone structure in the spine and weight-bearing bones (such as the hip) cannot withstand the force of gravity. Thus, patients with weakened bone structure from osteoporosis or metastatic cancer are at risk for painful fractures of the spine and hip. In addition, spine fractures place patients at risk for spinal deformity that prevents one’s ability to breathe and leads to early satiety, blood clots from inactivity, blood stasis lung infections, and increased risk of death (Berenson et al., 2011; Lipton, 2007).

When they were first introduced, clinicians welcomed the benefits of IV bisphosphonates in preventing SREs in patients with cancer. Bisphosphonates carried few common side effects such as acute phase reactions (transient fever, chills, or flu-like symptoms and hypocalcemia), which led to their widespread use and acceptance within the nursing and medical communities. Studies demonstrated improved quality of life, as compared with previous years, when patients with skeletal fractures experienced a shortened overall lifespan (Diel et al., 2004). This remains true, as several studies agree that when given bisphosphonates, patients develop fewer fractures, live longer, and have improved quality of life if treatment complications are not encountered (Kyle et al., 2007; Miksad et al., 2011; Palumbo et al., 2011).

It became evident in the late 1990s that bisphosphonates were not metabolized by the body. Most IV bisphosphonates are retained in the bone for extended periods of time, for up to several years (Ruggiero et al., 2009). For many types of cancers, overall survival has increased significantly. Thus, bisphosphonate metabolism and duration of bone absorption have become important considerations throughout one's lifespan. However, with several years of exposure, an unfortunate and very serious side effect of bisphosphonate therapy emerged.

## Initial Reports of BRONJ

Oral surgeons were among the first to notice an increase in referrals for jaw pain and nonhealing ulcers as early as 2001, yet the first cases of BRONJ were reported in a letter to the editor in *The Journal of Oral and Maxillofacial Surgery* in 2003 (Marx, 2003; Ruggiero, 2009). In his letter, Robert E. Marx, a surgeon at the University of Miami School of Dentistry, Oral and Maxillofacial Surgery Division, described 36 patients with bone exposure in the mandible and/or maxilla. All 36 patients were receiving either pamidronate or zoledronic acid. It was observed that 28 of the bone exposures were initiated by tooth removal, but the remaining 8 developed exposed bone without tooth removal (Marx, 2003).

**Figure 1:**
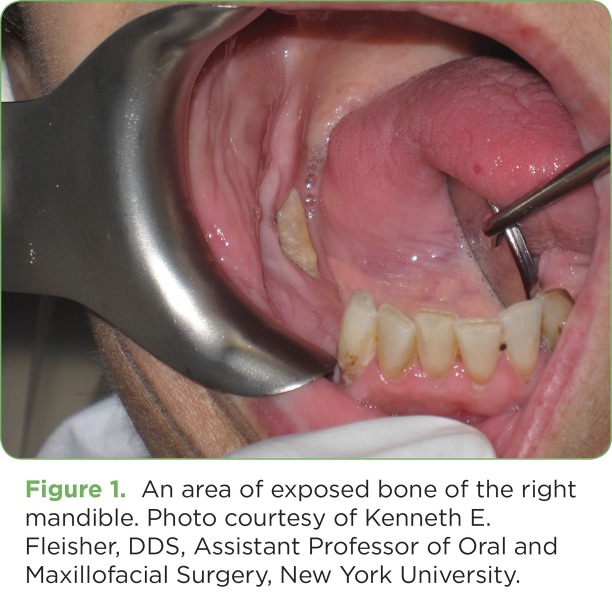
Figure 1. An area of exposed bone of the right mandible. Photo courtesy of Kenneth E. Fleisher, DDS, Assistant Professor of Oral and Maxillofacial Surgery, New York University.

This first report of BRONJ raised many concerns for patients and practitioners. Were bisphosphonates safe when given to humans? What was BRONJ, and did bisphosphonates cause the jaw pain, exposed bone (Figure 1), and poor healing described by Dr. Marx and in the dental literature? Bisphosphonate-related osteonecrosis was a problem that could plague patients with osteoporosis or rare bone disorders. Dentists had described two cases of mucosal infections underneath the dentures in patients undergoing cancer chemotherapy before the era of bisphosphonates (Schwartz, 1982). Thus in the early years, experts believed that chemotherapy patients experienced osteonecrosis of the jaw due to an interaction with chemotherapy. Jaw necrosis was not observed in nonchemotherapy patients.

## Legal and Ethical Issues: The MedWatch System

The FDA is responsible for ensuring the safety and efficacy of all regulated marketed medical products in the United States. The MedWatch system allows clinicians to report suspected adverse events pertaining to human drugs, medical devices, vaccines and biologics, dietary supplements, and cosmetics (US FDA, 2012). These adverse events may occur within the context of a clinical trial or following FDA approval of the agent in everyday clinical practice. The benefit of the MedWatch system is that when it is used, postmarketing information can be learned. Practitioners can file drug safety reports by phone, Internet, or fax to the FDA. This process can in turn lead to new safety information.

Based on MedWatch reports surrounding bisphosphonates in the early 2000s, a board of independent reviewers evaluated patient charts from two centers with a high incidence of jaw necrosis: Long Island Jewish Medical Center and the University of Miami. Data from bisphosphonate trials in cancer patients were reviewed to determine if bisphosphonates caused jaw necrosis. Following a thorough review, the committee affirmed that jaw necrosis was not a complication in any bisphosphonate trial but rather that it was a late side effect of chemotherapy (Ruggiero, 2009). Despite the absence of causality and the reluctance to associate bisphosphonates with jaw necrosis, in accordance with drug safety regulations in the United States, Novartis sent a "Dear Healthcare Professional" letter to oncology practitioners in 2004. This letter notified recipients of the possible association between bisphosphonates and jaw necrosis.

Additional case reports of jaw necrosis emerged in the literature (Badros et al., 2006; Kut, Mehta, Tariman, Olsson, & Singhal, 2004; Ruggiero, Mehrotra, Rosenberg, & Engroff, 2004). Warnings were added to the "Postmarketing Experience and Precaution" section of bisphosphonate package inserts to alert the patients and prescribers to this complication. Guidelines were created by multidisciplinary teams of experts as to the frequency of administration and how patients should be monitored when receiving bisphosphonates. Dentists and oral surgeons created peer-reviewed position statements to inform their practitioners of how to manage patients receiving dental procedures and the associated risks (Hillner et al., 2003; Kyle et al., 2007; Lacy et al., 2006; Mehrotra & Ruggiero, 2006). Experts contended that patients with cancer were living longer than in previous years due to better supportive care and more effective chemotherapy treatments. Therefore, many believed BRONJ was a late side effect of chemotherapy treatment rather than a drug class effect.

By 2006, 3 years after the initial concerns of jaw necrosis emerged, over 3,700 additional reports were made through the MedWatch system. As a result, the American Association of Oral and Maxillofacial Surgeons created a position paper on BRONJ (US FDA, 2007; Mehrotra & Ruggiero, 2006). Most MedWatch reports were a result of pharmacovigilance, the practitioner’s legal and ethical obligation to report adverse effects or any other drug-related problem (World Health Organization, 2011). Pharmacovigilance involves the measures that have been taken to raise awareness of prevention and treatment on behalf of drug manufacturers, physicians, and practitioners, as well as risk management from preclinical development stage to the postmarketing stage. Without such safety reporting, the phenomenon of BRONJ would not be understood.

## Epidemiology of BRONJ

Osteonecrosis of the jaw was unknown until about 10 years ago. The incidence of BRONJ in patients with a history of bisphosphonate use is debatable (Rizzoli et al., 2008; Albu & Dinu, 2010), as most of the data available today are limited to case reports, retrospective analyses, and patient surveys (Durie, 2007). The incidence remains nebulous, as there are no consistent reporting scales, and until recently, consensus criteria for the diagnosis of BRONJ were nonexistent.

The risk of BRONJ depends on both the potency and duration of bisphosphonate therapy (Durie, Katz, & Crowley, 2005). A case series of 252 patients with bone metastasis and the occurrence of BRONJ found that those who developed BRONJ had received a median of 35 infusions of IV bisphosphonates (pamidronate or zoledronic acid), while those who did not develop BRONJ had received a median of 15 infusions (Bamias et al., 2005). Approximately 5% of all patients treated with bisphosphonates for metastatic cancer are said to suffer from BRONJ later in life (Allen, 2011; Yamashita, McCauley, & Van Poznak, 2010); 10% to 20% of animals treated with bisphosphonates as part of various studies also develop some sort of necrosis of the jaw (Allen, 2011).

The frequency of BRONJ in cancer patients treated with IV bisphosphonates ranges from 0.7% (Hoff et al., 2008) to 12% (Ortega et al., 2007). The cumulative incidence ranges from 4.8% (Hoff et al., 2008) in breast cancer patients treated with IV bisphosphonates for more than 5 years to 40% (Dodson, 2009; Tosi et al., 2006) in multiple myeloma patients treated with IV bisphosphonates for 36 months. Other studies have shown an incidence of 6.8% to 9.9% in multiple myeloma patients and 2.9% to 4.4% in breast cancer patients (Bamias et al., 2005; Durie et al., 2005). In an analysis of 626 published cases of BRONJ, the incidence is highest for patients who received zoledronic acid followed by pamidronate and remains higher in patients with breast cancer and multiple myeloma (Reid, 2009). Oral clodronate, which is not available in the United States, carries a lower risk of BRONJ (Reid, 2009).

In a phase III randomized placebo-controlled clinical trial comparing 4 mg IV zoledronic acid and 120 mg SC denosumab (a nonbisphosphonate monoclonal antibody) in patients with metastases from multiple myeloma and other solid tumors except breast and prostate cancers, ONJ occurred with equal frequency in both groups (Henry et al., 2011). Another phase III trial comparing 4 mg IV zoledronic acid and 120 mg SC denosumab in patients with metastatic breast cancer also showed that ONJ was similar in both groups (Stopeck et al., 2009). Although denosumab is not considered a bisphosphonate, knowledge of the risk of ONJ must be conveyed to patients who begin treatment with this agent.

Other studies have evaluated BRONJ risk with oral agents. A nested case control study found an increased relative risk of osteonecrosis at any site among bisphosphonate users, with an adjusted relative risk of 2.87 for alendronate, 2.43 for etidronate, and 3.34 for risedronate (Khan, 2008). There are no proven reports of ONJ in patients who have taken bisphosphonates for osteoporosis (Khan, 2008a). The incidence of ONJ after 3 years was similar in the zoledronate and placebo groups in a study conducted to evaluate its incidence in patients with osteoporosis taking IV zoledronate to prevent fractures (Black et al., 2007). Postmarketing data of the orally administered amino bisphosphonates alendronate and risedronate suggest that the incidence is less than 1 case per 100,000 patients (Khan, 2008a). However, the risk of ONJ is higher with amino bisphosphonates than with non–amino bisphosphonates (Almazrooa & Woo, 2009).

Osteonecrosis significantly decreases the quality of life. This decrease is proportionate to the worsening of osteonecrosis (Miksad et al., 2011). The population at risk of ONJ associated with bisphosphonate treatment is sizeable. As of 2004, more than 3 million individuals worldwide had received bisphosphonate treatment (Miksad et al., 2011). Lifestyle factors such as the use of alcohol and tobacco (Almazrooa & Woo, 2009) as well as other medications that reduce osteoclastic activity (Yamashita et al., 2010) and glucocorticoids (Reid, 2009) are said to add to the risk of developing BRONJ. Bevacizumab (Avastin), an anti–vascular endothelial growth factor monoclonal antibody used in the treatment of various cancers, is also said to increase the risk of BRONJ with concomitant zoledronic acid use, but this is debatable (Aragon-Ching et al., 2009).

The negative impact of bisphosphonates and BRONJ should be weighed against the potential benefits when the advanced practitioner evaluates each patient (Miksad et al., 2011). Based on expert opinion, as the incidence of BRONJ is extremely low in bisphosphonate users, the incidence may be similar in the general population (Reid, 2009). In addition, since many other concomitant factors have been implicated in causing ONJ, the attributable risk of ONJ due to bisphosphonates is uncertain (Almazrooa & Woo, 2009; Ortega et al., 2007; Yamashita et al., 2010).

## Risk Factors

Several risk factors are associated with BRONJ. These include local, drug-related factors, demographics, and systemic factors. Among the local risk factors, some surgical procedures confer a seven times higher risk of developing BRONJ: dental extractions, implant placement, and periodontal surgery for severe periodontitis. Other local risk factors are represented by certain aspects of the local anatomy. Thus, BRONJ is more frequent in the mandible compared to the maxilla (2:1) and in the areas where exostosis, maxillary and mandible torus, and internal and external oblique ridges are present and covered by a thin mucosa. This occurs in patients with inflammatory and infective oral diseases such as dental abscesses and active periodontal pockets, and also presents a seven times higher risk for BRONJ. Although BRONJ usually occurs following surgical procedures of the jaw (including dental extractions), idiopathic cases have also been reported (Australian Ministry of Health, 2010).

Drug-related risk factors include the type of bisphosphonate, treatment duration, route of administration, and other associated drugs. The third-generation bisphosphonates (amino bisphosphonates), which are the most potent, induce a higher risk for BRONJ that increases after 1 year of administration. The longer the duration of treatment with bisphosphonates, the higher the risk of developing BRONJ. Regarding the route of administration, BRONJ risk is stratified: lower for the oral bisphosphonates and higher for the IV formulations (such as zoledronic acid and pamidronate).

## Prevention

Few studies surrounding the prevention of BRONJ have been conducted. The use of oral antibiotics for BRONJ has been studied in a retrospective study by the Italian Myeloma Group. Patients who required bisphosphonates and underwent dental procedures were randomized to receive antibiotic prophylaxis with amoxicillin-clavulanate 1 g twice daily by mouth or levofloxacin 500 mg/day by mouth if the patient was allergic to amoxicillin. Patients received antibiotics from 1 day before to 3 days after any dental procedure. Patients who received antibiotic prophylaxis surrounding dental procedures had a significant decrease risk (* p* = .012) to develop BRONJ. Thus, researchers concluded that antibiotic prophylaxis may prevent ONJ occurrence after dental procedures (Montefusco et al., 2008).

## Diagnostic Criteria and Treatment

Criteria for the diagnosis of BRONJ have been described (AAOMS, 2006). A patient without history of radiotherapy to the jaw may be considered to have BRONJ if there is evidence of (1) current or previous treatment with bisphosphonates, and (2) exposed, necrotic bone in the jaw area for a period of at least 2 months with no evidence of cancer at the site.

Treatment of BRONJ differs according to the stage of the disease. Table 1 provides information on staging, treatment, and implications for practitioners. Four main goals exist: (1) eliminate the active infection by administering systemic antibiotics, (2) control pain, (3) reduce the progression, and, if possible, (4) eliminate the occurrence of bone necrosis. Treatment consists of debridement of necrotic tissues, daily use of highly potent mouth rinses, and avoidance of future extensive surgical procedures. Chlorhexidine-based mouth rinses are preferable, as this antimicrobial substance is the "gold standard" in orodental surgical procedures. However, patients should be informed about the side effects of long-term use, such as taste changes, and offered an alternative treatment.

**Table 1:**
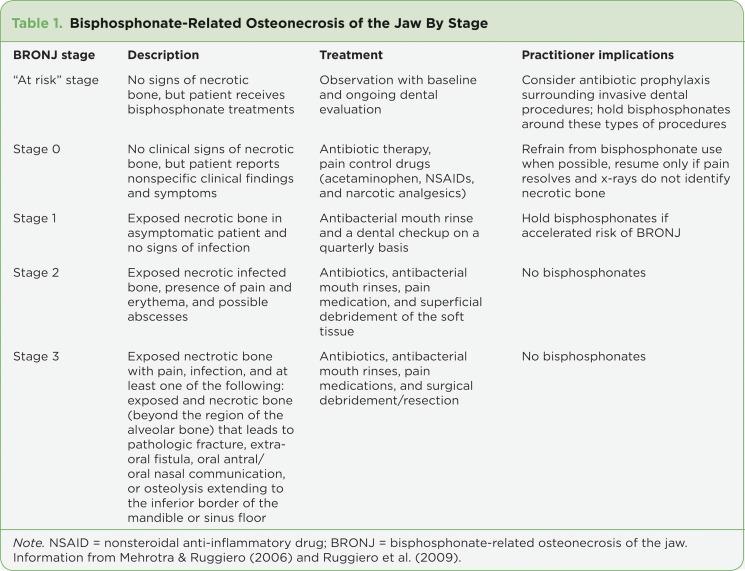
Table 1. Bisphosphonate-Related Osteonecrosis of the Jaw By Stage

## Role of the Advanced Practitioner

At baseline and throughout bisphosphonate treatment, the advanced practitioner must obtain a clear medical history, which includes both past and present medical issues. All patients should undergo a thorough oral health assessment and receive treatment for any pathologies in the orodental area before bisphosphonate administration. Necessary dental treatment can be continued up to the "window period," when the risk of developing BRONJ is low but still existent. Patients at risk for BRONJ should be properly monitored and undergo routine examinations after any surgical intervention in the orodental area after 1 week, 1 month, 3 months, 6 months, and 12 months.

The individual is considered to be at risk for developing BRONJ if bisphosphonate therapy has been received. Patients should be advised to notify all dental practitioners of prior bisphosphonate therapy. If the advanced practitioner notes any evidence of exposed bone or jaw pain, or pain in general, the patient should be referred to a dentist. Orodental examinations include evaluating the patient’s oral hygiene status, tooth decay, and active periodontitis as well as indicating oral education. In addition, the dentist and/or advanced practitioner should perform radiographies of the painful areas of interest.

## ETHICS IN RESEARCH AND PRESCRIPTION OF BISPHOSPHONATES

Many advanced practitioners prescribe bisphosphonates on a regular basis. Associated risks, benefits, and alternatives to treatment are discussed with patients. However, to further advance scientific knowledge, ongoing research with bisphosphonates is essential. Phase III randomized trials compare a new therapy with a "standard of care" regimen to determine the superiority of one regimen over another. The risks, benefits, and alternatives must be discussed with patients who participate in future clinical trials with bisphosphonates, as well as included in the informed consent document. According to the ClinicalTrials.gov database, there are 346 bisphosphonate trials open, with 87 actively recruiting. Examples of clinical studies open to accrual include those investigating the development of BRONJ risk assessment tools, the effect of bisphosphonates on oral soft-tissue wound healing, and a pilot trial of IV pamidronate for low-back pain.

## LEGAL CONSIDERATIONS

The advanced practitioner must be aware of the legal implications of bisphosphonate therapy, whether it is within the context of a clinical trial or not. Should a patient develop BRONJ, it is recommended to report it to the FDA through the MedWatch system. It is known that individuals who have participated in bisphosphonate trials or received these drugs outside of a clinical trial and developed BRONJ may wish to seek restitution.

Since BRONJ has been reported, a series of lawsuits have been filed by individuals who developed this rare complication. Many of the lawsuits have alleged that the complication was a result of taking zoledronic acid and/or pamidronate. A search of US federal and state court cases was performed in the LexisNexis Academic database. Using the search terms "bisphosphonate" and "osteonecrosis", 49 cases were found in various stages of litigation. Of these, four cases have reached the US Court of Appeals. Each of the four cases ruled in favor of Merck or Novartis in upholding the lower court decisions.

## Considerations for Future Research

Several clinical trials have been conducted to determine the safety and efficacy of bisphosphonates in osteoporosis and cancer patients (Licata, 2006; Rosen et al., 2003). Future trials involving bisphosphonate use are essential to improve knowledge and should be conducted. Strategies involve understanding the etiology of BRONJ thoroughly, taking care to avoid concomitant risk factors, and finding ways to prevent the development of BRONJ.

Many of us are unlikely to design and implement our own bisphosphonate trials. However, it is important to understand the risks, benefits, and alternatives to treatment with bisphosphonates, whether drugs are given within the context of a trial or not. Here, in Table 2, we delineate evidenced-based recommendations to be considered when initiating oral or IV bisphosphonate therapy. In addition, methodologic considerations for future research are listed in Table 3.

**Table 2:**
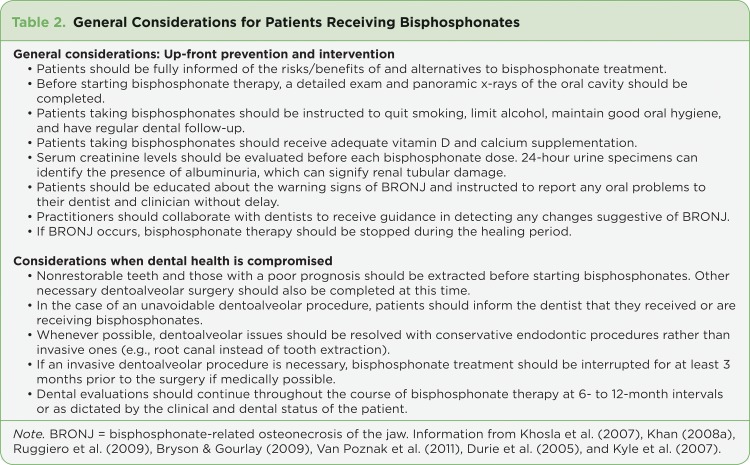
Table 2. General Considerations for Patients Receiving Bisphosphonates

**Table 3:**
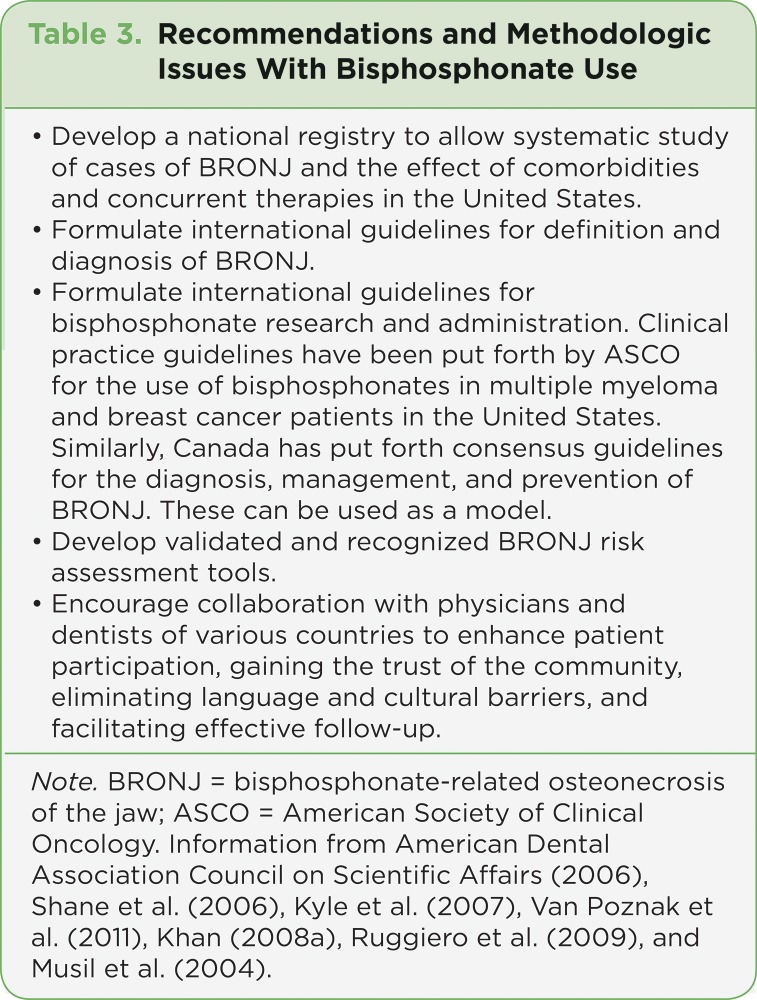
Table 3. Recommendations and Methodologic Issues With Bishosphonate Use

## Conclusion

Bisphosphonates provide patients with cancer the hope of decreased fracture risk and improved quality of life. Although BRONJ is a painful but rare side effect, the risk should be disclosed in future research and to patients who receive bisphosphonates or similar drugs for treatment of cancer-related bone disease. Many years have passed since the initial bisphosphonate trials were conducted in patients with cancer and osteoporosis. However, unanswered questions as to the causality, pathogenesis, and susceptibility to develop BRONJ remain. Data collection in regard to safety, dose, and duration of use is ongoing. Thus, future clinical trials should be conducted in a safe and ethical manner using recommendations that were cited within this article. Advanced practitioners should bear patient safety first and foremost in mind, but take all measures possible to protect themselves legally through clear disclosure of side effects and the use of safe prescribing practices.
